# The Anticipation of Gravity in Human Ballistic Movement

**DOI:** 10.3389/fphys.2021.614060

**Published:** 2021-03-17

**Authors:** Janice Waldvogel, Ramona Ritzmann, Kathrin Freyler, Michael Helm, Elena Monti, Kirsten Albracht, Benjamin Stäudle, Albert Gollhofer, Marco Narici

**Affiliations:** ^1^Department of Sport and Science, University of Freiburg, Freiburg, Germany; ^2^Department of Biomechanics, Rennbahnklinik, Muttenz, Switzerland; ^3^Neuromuscular Physiology Laboratory, Department of Biomedical Sciences, University of Padua, Padua, Italy; ^4^Faculty of Medical Engineering and Technomathematics, Aachen University of Applied Sciences, Aachen, Germany; ^5^Institute of Biomechanics and Orthopedics, German Sport University Cologne, Cologne, Germany; ^6^Institute of Movement and Neurosciences, German Sport University Cologne, Cologne, Germany; ^7^Myology Centre ‘CIR-Myo‘, Neuromuscular Physiology Laboratory, Department of Biomedical Sciences, University of Padua, Padua, Italy

**Keywords:** load, stretch-shortening cycle, neuro-mechanics, elastic energy, acceleration, muscle-tendon unit, electromyography

## Abstract

Stretch-shortening type actions are characterized by lengthening of the pre-activated muscle-tendon unit (MTU) in the eccentric phase immediately followed by muscle shortening. Under 1 g, pre-activity before and muscle activity after ground contact, scale muscle stiffness, which is crucial for the recoil properties of the MTU in the subsequent push-off. This study aimed to examine the neuro-mechanical coupling of the stretch-shortening cycle in response to gravity levels ranging from 0.1 to 2 g. During parabolic flights, 17 subjects performed drop jumps while electromyography (EMG) of the lower limb muscles was combined with ultrasound images of the gastrocnemius medialis, 2D kinematics and kinetics to depict changes in energy management and performance. Neuro-mechanical coupling in 1 g was characterized by high magnitudes of pre-activity and eccentric muscle activity allowing an isometric muscle behavior during ground contact. EMG during pre-activity and the concentric phase systematically increased from 0.1 to 1 g. Below 1 g the EMG in the eccentric phase was diminished, leading to muscle lengthening and reduced MTU stretches. Kinetic energy at take-off and performance were decreased compared to 1 g. Above 1 g, reduced EMG in the eccentric phase was accompanied by large MTU and muscle stretch, increased joint flexion amplitudes, energy loss and reduced performance. The energy outcome function established by linear mixed model reveals that the central nervous system regulates the extensor muscles phase- and load-specifically. In conclusion, neuro-mechanical coupling appears to be optimized in 1 g. Below 1 g, the energy outcome is compromised by reduced muscle stiffness. Above 1 g, loading progressively induces muscle lengthening, thus facilitating energy dissipation.

## Introduction

A highly exciting field of research of reactive movements is the mode of energy transfer and thus energy management during various movements and loading conditions (Lindstedt et al., 2001; Arampatzis et al., 2004). During ideal reactive movements kinetic energy (E_kin_), which a human has by virtue of its movement prior to touchdown, can be stored during ground contact and subsequently released at take-off (Komi and Bosco, 1978; Cavagna et al., 1985; Bosco et al., 1987; Komi, 2000). Therefore, the quality of energy management can be determined by the net kinetic energy between touch down and take-off and the amount of kinetic energy at take-off.

It is well-established that an early and high pre-activity and reflex activity of the leg extensors is the prerequisite to optimize the energy turnover to store or absorb energy in the MTU (Avela et al., 1996; Komi and Gollhofer, 1997). Thereby precise neuro-mechanical coupling is essential for efficient energy management which determines the economy of movement for minimizing metabolic energy expenditure while maximizing power output for the performance enhancement (Komi, 2000; Arampatzis et al., 2001). Particularly, precise pre-activity of motor units in the agonistic muscles of the lower limbs is required to ensure locomotion like running or jumping safely. The functional purpose of the pre-activity is the stiffening of muscles to stabilize joints in order to protect the musculoskeletal structures from impact forces upon landing. This enables tendons to store elastic energy, subsequently released in the concentric phase of the stretch-shortening cycle. This reduces the time required to develop high forces and enables a quick contraction and movement (Arampatzis et al., 2001; Finni et al., 2001). Also, the net energy outcome in the stretch-shortening cycle depends on the recoil properties of the MTU as well as the fascicle shortening and lengthening behavior, which is determined by the muscle's level of activity before and during touch-down. Tendons operate in series with the muscles and can only act spring-like when the muscles themselves generate force. Elongation and shortening of the MTU are mainly induced by length changes in the tendinous tissue, whereas length of muscle itself only marginally change as evidenced by ultrasound measurements in human walking (Fukunaga et al., 2001; Lichtwark et al., 2007) and jumping (Ishikawa et al., 2003; Sousa et al., 2007). It has been demonstrated that optimal energy management requires quasi-isometric muscle contraction during the eccentric phase of the GC phase to enable exclusive length changes in the tendinous structures (Hirayama et al., 2017).

Both muscle activity and the recoil properties of the MTU are load-dependent. Up to an individual load tolerance this dependency is proportional to the ground reaction forces (Komi and Bosco, 1978; Schmidtbleicher and Gollhofer, 1982; Ishikawa et al., 2003). Based on major motor control theories, it is evident that neuro-mechanical control in locomotive movement on Earth is perfectly tuned to the constant 1 g gravity force vector. As the humans' body mass remains constant in alternate gravitational conditions like in space, acceleration induced joint moments are the major variable determining the energy management involving storage or dissipation of kinetic energy (Lindstedt et al., 2001; Gambelli et al., 2016). Thus, modification of the acceleration such as an increase in gravity (>1 g) is expected to require an immediate increase in muscle activity and muscle force production (Gollhofer and Kyröläinen, 1991; Bubeck and Gollhofer, 2001; Kramer et al., 2012). Vice versa, a decrease in the gravitational force (<1 g) is considered to cause a reduction in neuromuscular performance (Gollhofer and Kyröläinen, 1991; Avela et al., 1996). Moreover, in addition to long-term gravity-dependent modulations, sudden changes in gravity require anticipatory changes of the neuromuscular system according to the loading (Avela et al., 1996). Hence, it is evident that changes in the body's loading considerably affect motor actions during motion. This is important because external loading (variable gravity) affects both the kinematics and the muscle activity accordingly.

Phase- and load-specific modulations in ballistic movements have intensively been investigated in the past by using different falling heights, added mass or sledge jump systems (Gollhofer and Kyröläinen, 1991; Gollhofer et al., 1992; Avela et al., 1994; Bubeck and Gollhofer, 2001). However, especially very low and very high kinetic energy conditions are challenging and require an artificial test setup which is naturally accompanied by confounding effects (Avela et al., 1996; Kramer et al., 2012). Additionally, empirical evidence on how muscle activity is systematically modulated within a wide range of acceleration or gravity profiles during stretch-shortening cycle actions is still limited. Further, the specific dependency of the MTU and effective length changes of the muscle fascicles, energy management and functional performance has not been investigated yet.

Therefore, this study aimed to identify load-dependent changes in neuromuscular and biomechanical characteristics of the stretch-shortening cycle in drop jumps as a function of gravitational loading concerning 1 g. We hypothesized that (1) phase-specific modulations in muscle activity would be accompanied by biomechanically relevant kinematic adaptations in response to gravity variation. Thereby, (2) leg muscle activity prior and during GC would subsequently affect the neuro-mechanical coupling of the MTU resulting in (3) altered net energy outcome, and relevant SCC performance indicators.

## Materials and Methods

### Subjects

Seventeen subjects (7 female, 10 male, height 175 ± 9 cm, body mass 72 ± 12 kg, age 31 ± 5 years) participated in this study. All participants gave written informed consent to the experimental procedure following the latest revision of the Declaration of Helsinki that was approved by the French authorities responsible for the protection of subjects participating in biomedical research (DEMEB of the AFSSAPS) and the Ethics Committee of the University of Freiburg (430/17). The participants underwent two medical investigations to verify eligibility for the experiment. Exclusion criteria were pregnancy, sickness, neurologic or orthopedic injuries, vestibular or proprioceptive dysfunction, fear of flying, previous surgeries on the left or right leg, neurodegenerative diseases or single events associated with neural dysfunctions. Inclusion criteria were reliable reactive jump pattern and experience in parabolic flights (≥ one flight).

### Experimental Design

**Figure 1:**
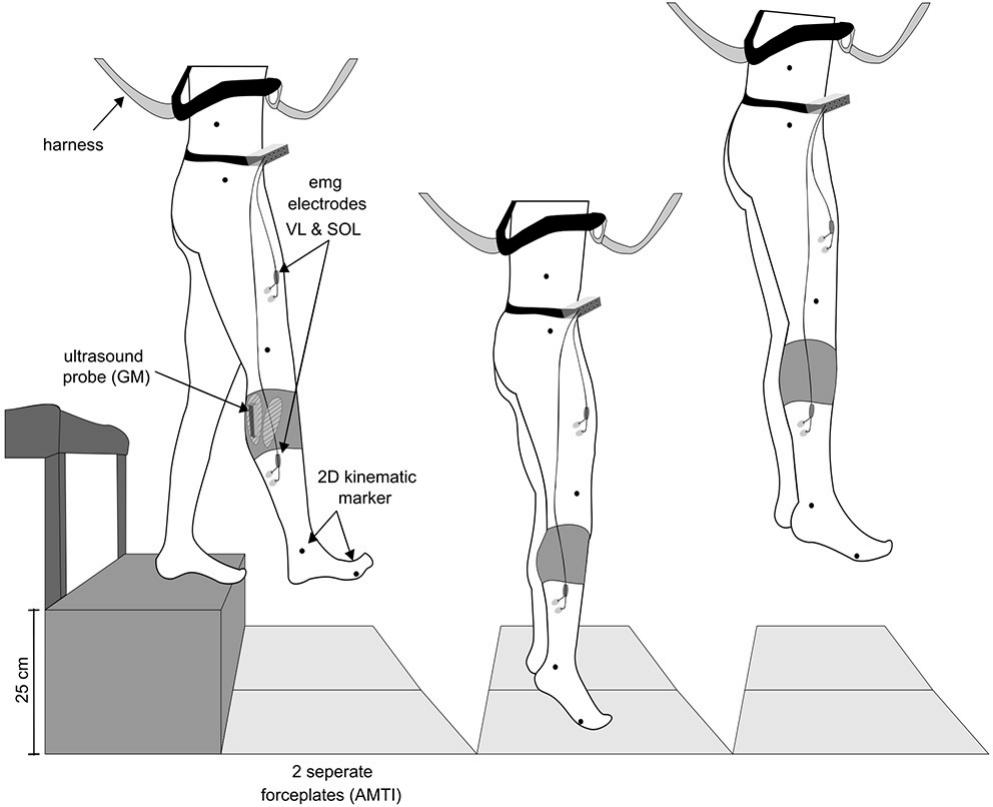
Schematic overview of the experimental setup. Subjects performed drop jumps from a rack with a drop height of 25 cm to (1) separated AMTI force plates. Additionally, they were safely attached to (2) a harness during the whole flight. Electromyographic activity (EMG) of the shank and thigh muscles were recorded using (3) Ag/CL electrodes, shown for the rectus femoris (RF) and soleus (SOL) as an example. For monitoring the gastrocnemius medialis' (GM) behavior (4) a linear ultrasound probe was placed on the muscle belly and secured with a unique device in order to avoid probe's movements against the skin. (5) 2D kinematic markers were placed on the iliac crest, trochanter major, lateral condyle of the knee joint, malleolus and the 5th metatarsal bone.

**Figure 2:**
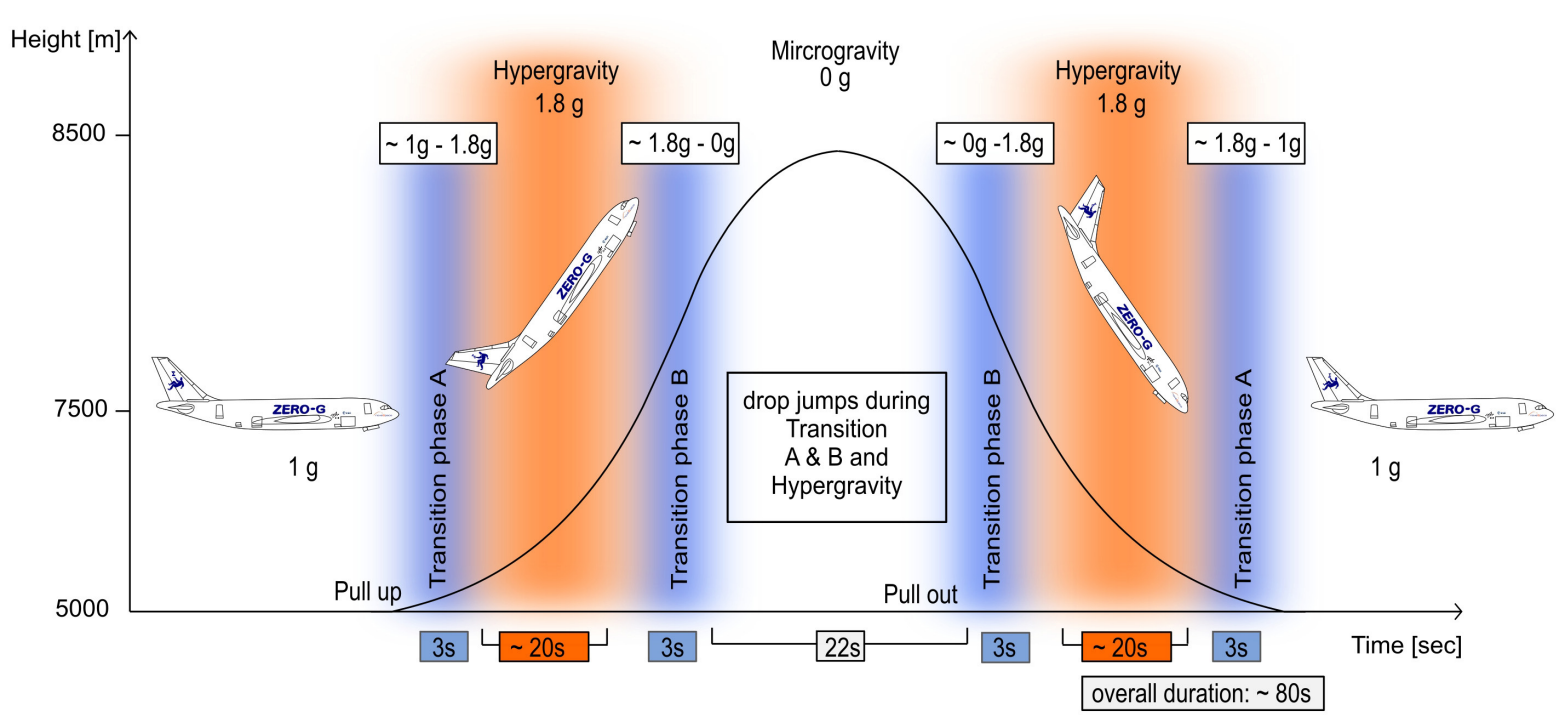
Parabolic flight maneuver and the corresponding gravity levels. Steady flight (1 g) is followed by a pull-up phase inducing hyper-gravity (ca. 20 s), followed by zero gravity (22 s) and another hyper-gravity phase (ca. 20 s) before returning to a level of normal gravity flight (1 g). The hyper-gravity phases (orange) and the transition phases A and B (blue) were used to assess neuro-mechanics in the stretch-shortening cycle during jumping; reference jumps were performed during 1 g.

A single-group repeated-measures study design was used to examine differences between drop jumps performed in 1 g with drop jumps performed below and above 1 g based on the recorded ground reaction forces, kinematics, electromyographic activity of the lower limb muscles and the muscle and MTU behavior of the gastrocnemius medialis (GM). The experimental setup is illustrated in Figure 1. The reference measurements in the 1 g condition were recorded on ground; in two subjects reference measurements were performed during steady flight. During parabolic flight maneuvers (Figure 2), the jumps were clustered into nine progressively increasing gravity bounds as follows: 0–0.25, 0.25–0.5, 0.5–0.75, 0.75–1.0, 1.0, 1.0–1.25, 1.5–1.75, and 1.75–2.0g. The order of the different gravity levels was pseudo randomized between subjects to control for confounding effects like habituation; fatigue was avoided by the rest periods in between the parabolas and sets of jumps, respectively (~2 min).

After a 10-min warm-up (running, tappings, and bouncing executed in the aircraft) and familiarization according to Kramer et al. (2010), subjects performed reactive drop jumps barefoot from a drop height of 25 cm with the instruction to jump stiff with knee and hip joints almost extended and to keep the ground contact time as short as possible, ensuring the most reliable jump pattern (Ritzmann et al., 2016). In order to protect the participants from injuries of the musculoskeletal apparatus and to avoid learning effects during the experiment, the subjects performed a preconditioning training before the campaign (4 weeks/3 times a week). Training consisted of reactive drop jumps with and without additional weight; the load was progressively and individually increased during the training period.

### Parabolic Flights and g-Level Cluster

The experiments were conducted aboard the ZERO-G aircraft (operated by Novespace, Bordeaux, France) during the 69th and 70th ESA and 33th DLR parabolic flight campaign (PFC). The campaigns included three flight days; each flight lasted 3 h and comprised 31 zero gravity parabolas. The course of one parabola is illustrated in Figure 2: each parabola includes a 22 s zero gravity (0 g) period embedded by two hyper-gravity (~1.8–2 g) periods of 20 s (Kramer et al., 2013). To assess the neuro-mechanics in the stretch-shortening cycle in varying gravity levels, the two hyper-gravity and four transition phases of each parabola were used (Figure 2). In total 1,236 jumps were analyzed; between gravity conditions jump number varied between 44 and 278 jumps per gravity condition.

The acceleration data (x, y, and z component) was recorded with an accelerometer, placed at the experimental rack. To cluster the gravity levels, the following criteria were applied: the exact g-level was defined for three discrete time points and two time intervals (150 ms before ground contact, instant of GC, instant of take-off, time interval from GC until TO and time interval 150 ms before GC until TO) based on the acceleration along the axis perpendicular to the floor of the plane. Trials were eliminated if vibrations or turbulences occurred. Inconsistent gravity levels characterized by high deviations during the jumps (above 0.3 g) were excluded from further analysis. This ensured that only conditions within upper or lower predefined boundaries as well as smooth trajectories without vibrations were included. Within the time interval from 150 ms before GC until take-off, the nine gravity clusters mentioned above contain the following means and standard deviations: 0.19 ± 0.06, 0.39 ± 0.08, 0.62 ± 0.08, 0.91 ± 0.07, 1.00 ± 0.05, 1.11 ± 0.08, 1.41 ± 0.07, 1.64 ± 0.08, and 1.81 ± 0.05.

### Outcome Measures

#### Force Measures

Ground reaction forces (GRF) for the left and right leg were recorded with a separated AMTI force plate (OR6-6, AMTI, Watertown, USA) with a sampling frequency of 2 kHz.

#### Electromyography (EMG)

Bipolar Ag/AgCl surface electrodes (Ambu Blue Sensor P, Ballerup, Denmark, diameter 9 mm, center-to-center distance 34 mm) were placed over the biceps femoris (BF), rectus femoris (RF), vastus medialis (VM), tibialis anterior (TA), gastrocnemius medialis (GM), gastrocnemius lateralis (GL) and soleus (SOL) muscles of the right leg. The longitudinal axes of the electrodes were in line with the presumed direction of the underlying muscle fibers at rest. The reference electrode was placed on the lower part of the tibia. Inter-electrode resistance was recorded by a multimeter and was kept below 5 kΩ using shaving, light abrasion and degreasing of the skin with a disinfectant. Procedures were executed according to SENIAM (Hermens et al., 2000). The EMG signals were transmitted via shielded cables to the amplifier (band-pass filter 20 Hz to 1 kHz, 200 × amplified) and recorded with 2 kHz (A/D-conversion via a National Instruments PCI-6229 DAQ-card, 16 bit resolution). Before the flights, subjects performed isometric maximal voluntary contractions (MVC) for each recorded muscle as well as countermovement jumps (CMJ). Highest EMG values were used for data normalization per subject (EMG maximum peak ± 25 ms). The MVCs were executed according to Roelants et al. (2006) and Wiley and Damiano (1998) performed isometrically (at joint level) against resistance and held for 3 s with 1 min in between. Body position during MVCs was strictly controlled and standardized using supervision by the authors. The EMG electrodes stood in place during MVC testing, reference measurements in 1 g and during the experimental conditions during the parabolic flights.

#### Ultrasonography

Muscle fascicles of the GM were imaged using B-mode ultrasound with a capture frequency of 82 Hz. The transducer was positioned over the medial part of the GM muscle belly to visualize fascicles and aponeuroses according to Werkhausen et al. (2017). The transducer (96-element, 6 cm linear-array probe, B-mode, frequency of 7 MHz, imaging depth of 50 mm and width of 60 mm) was securely fastened to the skin with adhesive tape at the interface of a very light plastic frame, built by a 3D printer, to avoid probe movement above the skin during jumping. The ultrasound data were recorded with Telemed Echo Wave II—Vers. 3.6.2 (Telemed ltd., Lithuania). Fascicle length and pennation angle were analyzed for 100 ms before GC until take-off, ECC (GC until minimum ankle joint) and CON (minimum ankle joint until take-off) by using a semi-automated tracking algorithm developed in MATLAB R2019A (Math Works, Natick, United States) and extrapolated where necessary (Thomas et al., 2015). The used tracking algorithm has been validated by Ultra Track (Farris and Lichtwark, 2016) (*r* = 0.66) and by analyzing the same jumps manually with ImageJ. Intraclass Correlation Coefficient (ICC) indicate good reliability between the two analysis methods (ICC = 0.83).

#### Kinematics

The 2D kinematics of the right limb segments were recorded with a high speed camera (Basler ace acA1920, Basler AG., Ahrensburg, sampling frequency of 100 Hz), placed at a distance of 1 m from the force platform, perpendicular to the sagittal plane (Gambelli et al., 2016). Markers were taped on the participants' skin on the following anatomical landmarks from top to bottom (Figure 1): iliac crest, greater trochanter, lateral femoral condyle, lateral malleolus, and fifth metatarsal (Gambelli et al., 2016). SIMI Motion software (SIMI Reality Motions System GmbH, 85716 Unterschleißheim, Germany) was used for the kinematic recording and analyses.

#### Data Processing

The effect of gravity on the force plate was accounted by resetting the GRF before GC. GRFs were used to determine peak forces normalized to body mass (F_max/mass_ in order to account for differences between subjects. Furthermore, jump height (JH), ground contact time (GCT; the time interval between GC and take-off) and reactive strength index (RSI, JH divided by GCT) were calculated. Regarding the experimental setup, the flight time method for JH calculation was not appropriate, as the indoor height of the airplane externally limited the JH. Therefore, especially below 1 g (JH would have exceeded the top of the airplane's ceiling), the operators kept hold of the harness to protect subjects from injuries and the airplane interior from damage. Iliac crest marker was tracked and assumed to represent the center of mass (COM); the COM's trace was used to calculate the vertical displacement of the marker during the GC phase, further reported as COM displacement. The first derivative of the pelvis marker was calculated to determine the COM velocity at take-off (v_to_). JH was then calculated by the following equation:

(1) $$\begin{array}{l} \text{J}\text{H}=\frac{{{\text{v}}_{\text{t}\text{o}}}^{2}}{2\text{g}}+h_{0} \end{array}$$

The corresponding mean value of gravity (g) was calculated for the propulsive phase (turning point eccentric/concentric: COM velocity = 0 until take-off). In order to account for the COM shift at take-off compared to the reference COM in the upright standing, the COM position was approximated by the iliac crest marker during the upright standing in 1 g. h_0_ corresponds to the difference of the COM shift from upright standing until take off.

The threshold for GC and take-off was set to 20 Newton.

The EMG activity was rectified, averaged, integrated (iEMG) and time normalized (to 1 s) for the following phases: pre-activity (PRE, 100 ms prior GC), eccentric (ECC, GC to minimal ankle angle) and concentric phase (CON, minimal ankle angle until take-off). Afterwards, iEMG was normalized to the MVC of the corresponding muscle.

Ankle and knee joint angles were determined for the event of GC and maximum flexion amplitudes [°] were calculated from GC until COM reached its minimum. For the ultrasound data, minimum ankle angle was used to divide the GCT into ECC and CON phase of the stretch-shortening cycle (Hoffrén et al., 2011). The kinetic energy was calculated for the time points, one frame prior to GC (E_kin_ GC) and take-off (E_kin_ TO) based on the iliac crest marker's velocity (COM) via the formula:

(2) $$\begin{array}{l} {\text{E}}_{\text{kin}}=0.5\;\text{m}{\text{v}}^{2}\; \end{array}$$

The following equation approximated the MTU's length (LMTU) according to Hawkins and Hull (1990) including β as the knee angle, θ as the ankle angle and L_Seg_ being the length of the shank segment:

(3) $$\begin{array}{l} {\text{L}}_{\text{mtu}}=\left[0.9+\left(-0.00062\;\beta \;\text{knee}\right)+\left(0.000214\;\theta \;\text{ankle}\right)\right]\;{\text{L}}_{\text{seg}}\; \end{array}$$

Fascicle length (L_f_) was defined as the length between the insertions to the superficial and deep aponeuroses. Pennation angle (α) was defined as the angle between fascicles and the deep aponeurosis. The effective length of the muscle (LM) and tendinous tissue (LTT) were calculated using the following formula:

(4) $$\begin{array}{l} {\text{L}}_{\text{M}}={\text{L}}_{\text{f}}*\text{ cos}(\text{α}) \end{array}$$

(5) $$\begin{array}{l} {\text{L}}_{\text{TT}}={\text{L}}_{\text{MTU}}-\;{\text{L}}_{\text{f}}*\text{ cos}(\text{α})\; \end{array}$$

The effective muscle length refers to the projection of the fascicle length in the longitudinal direction of the muscle (Fukunaga et al., 2001); importantly, effective muscle fascicle length changes do not refer to the whole length of the muscle fascicle. In order to synchronize the different methodological devices with the abovementioned recording frequencies, the whole data set was interpolated to 2 kHz. Python 3 was used for the analysis and the calculation of the parameters. All data were averaged for each gravity condition for each subject. Mean and standard deviations are given for the sample.

### Statistics

The statistical analysis was conducted using SPSS 24.0 (SPSS Inc., Chicago, Illinois) and Python 3. To evaluate the effect of gravity on the muscle activity, kinematics, kinetics, effective muscle and MTU behavior, a linear mixed model ANOVA (LMM) was used. Thereby the gravity was included as fixed, random and repeated factor. As the total amount of jumps varied per gravity condition jumps were also included as random and repeated factors. To account for intra- and inter-individual differences within and between subjects, subjects were also treated as random factor. The dependent variables included the muscle activity in PRE, ECC, CON for the shank and thigh muscles, as well as GCT, F_max/mass_, JH, reactive strength index, ankle and knee angles at GC, maximum flexion amplitudes of the ankle and knee joint and COM displacement during ECC; and absolute length changes of the MTU (LMTU), muscle (LM) and tendinous tissue (LTT) of the GM. The normality of the data was evaluated by q-q diagrams, histograms and boxplots. Data followed a normal distribution. The level of significance was set to *p* = 0.05. *T*-tests were used to compare the dependent variables in the gravity clusters below and above 1 g with the 1 g reference jumps. The false discovery rate was controlled according to the Benjamini-Hochberg-Yekutieli method as a rigorous statistical approach conceptualizing the rate of type I errors (Benjamini and Hochberg, 1995; Benjamini and Yekutieli, 2005). Eta square (η^2^) was used as an estimate of the effect size for the mixed model ANOVA [η^2^ ≤ 0.01 small, 0.01 ≥ η^2^ ≤ 0.14 medium, η^2^ ≥ 0.14 large effect size (Cohen, 1988)].

LMM was also calculated to model relative contribution of key parameters predicting net kinetic energy outcome. Thereby significant statistical interrelations between E_kin_ (DV, dependent variable), gravity (IV, independent variable), GM EMG PRE and ECC (IV) and COM displacement (IV) were proven. Thereby DV und IVs have been composed with reference to current scientific literature.

Additionally LMM was calculated to model the interrelations between F_max/mass_ (DV), gravity (IV) and ECC (IV). The goodness of fit was evaluated by Akaike-Information-Criterion (AIC) and root mean square error (RMSE) of the residuals. To get a brief understanding of the absolute model fit, marginal and conditioned RLMM (RLMM_m_, RLMM_cond_) was calculated (Nakagawa and Schielzeth, 2013). This is a general but simple method to estimate the variance explained by LMM; importantly, *R*^2^ commonly reported for linear regression analysis is not the same for LMM but RLMM_m_, RLMM_cond_ deliver an approximation of the explained variance by the model.

## Results

There were two participant dropouts due to motion sickness; data sets were excluded from analysis. Ultrasound data could not be analyzed from two subjects due to insufficient imaging quality as reliable detection of muscle fascicles was disabled.

**Figure 3:**
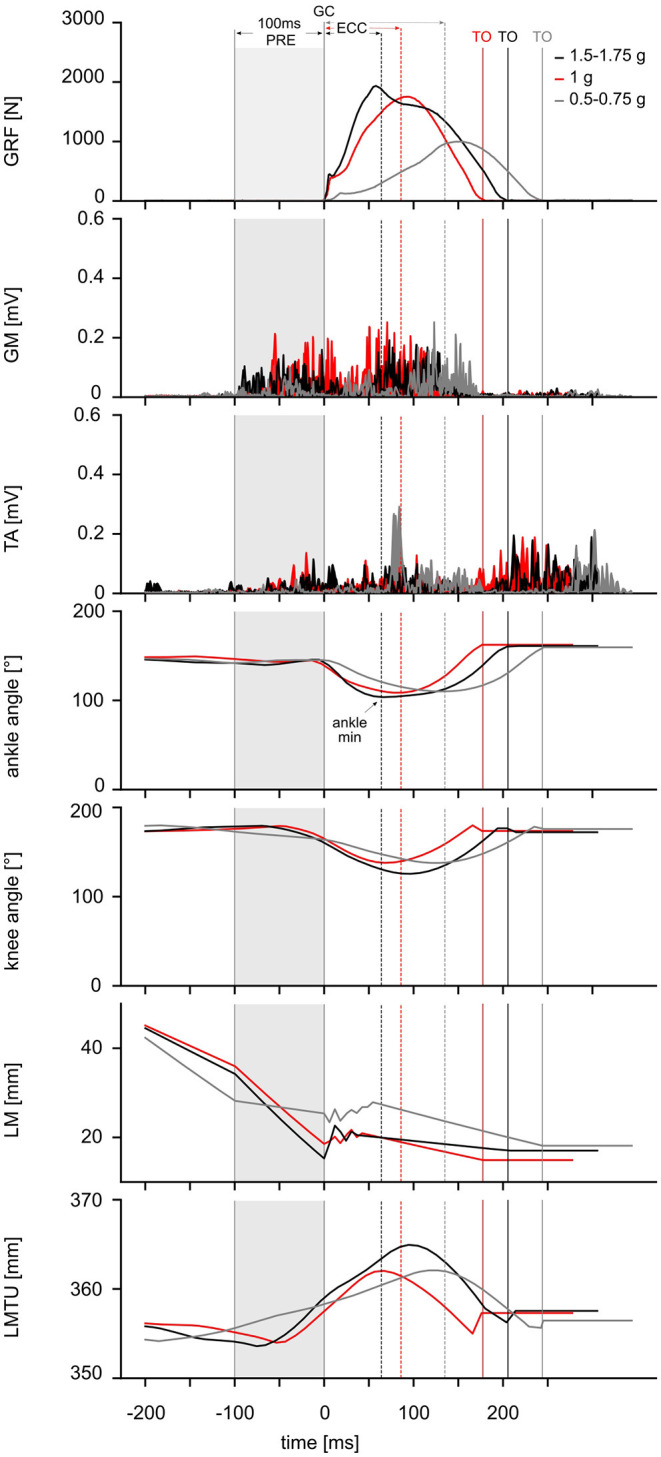
Experimental data from one representative subject. From the *top* to *bottom*: Modulation of ground reaction forces (GRF) (*top*), electromyographic activity (EMG) of the shank muscles [gastrocnemius medialis (GM), tibialis anterior (TA)], ankle and knee joint kinematics as well as length changes within the muscle [LM] and muscle-tendon unit [LMTU] (*bottom*) are illustrated as time course for one representative subject in response to three gravitational conditions: 0.5–0.75 g (*gray*), 1 g (*red*) and 1.5–1.75 g (*black*). Ground contact time (GCT) is marked as the time between initial ground contact (GC) and take-off (TO, also marked as *vertical solid lines* in the corresponding color). Additionally, following phases during the stretch-shortening cycle are marked with arrows (*top*): PRE [100 ms before GC until GC (*gray shadow*)] and ECC [GC until minimum ankle angle]. The CON phase is defined for the time interval between minimum ankle angle until TO. The time point when the ankle angle reached its minimum is marked as a *dashed line (*−−*)* in the corresponding color.

**Figure 4:**
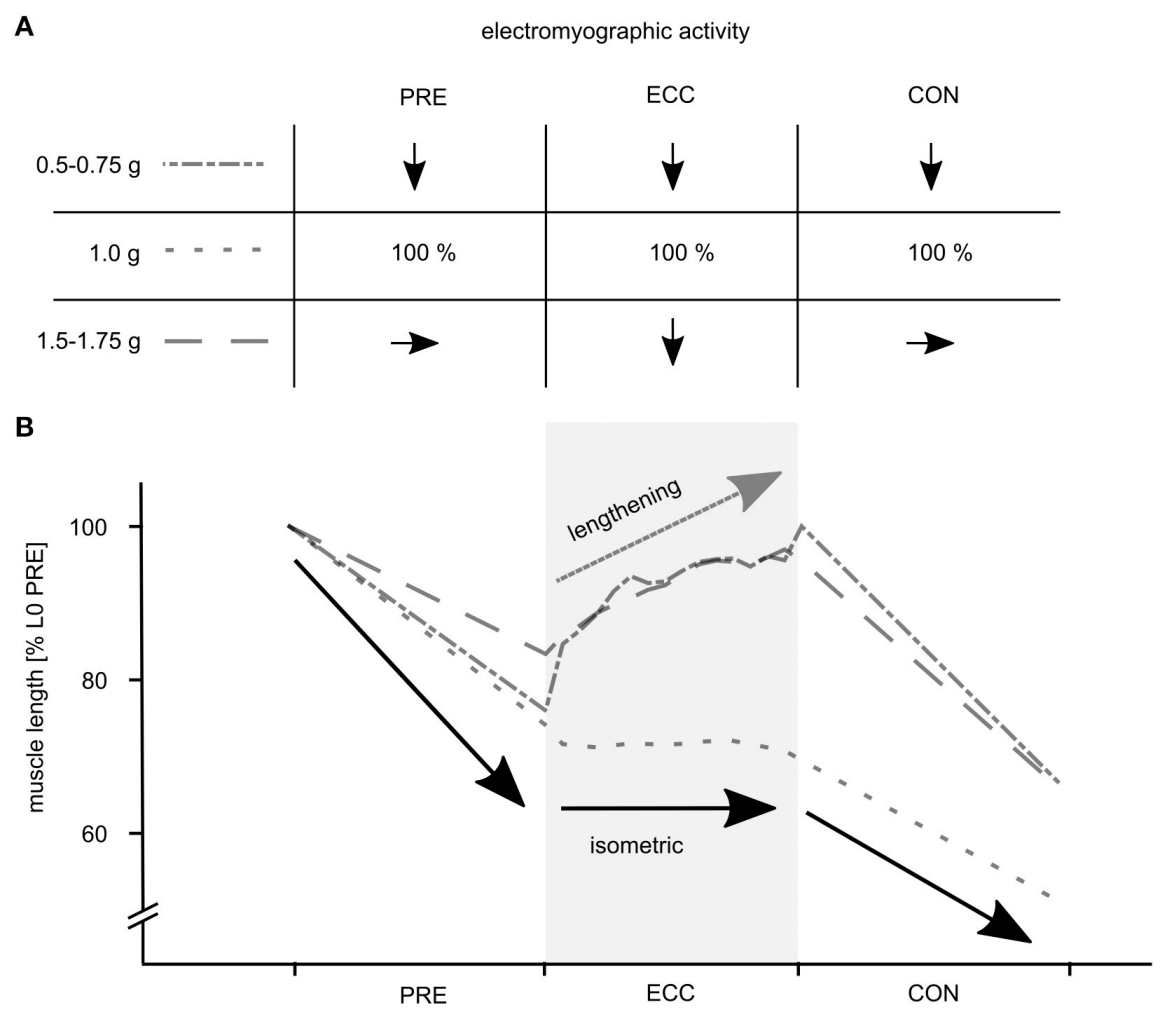
Mechanistic model of the phase specific muscle activity and the effective muscle behavior of the gastrocnemius medialis (GM) in response to variable acceleration levels determining the energy management during the stretch-shortening cycle. **(A)** The muscle activity is pre-programmed based on the afferent input. Compared to 1 g PRE EMG increases proportionally from 0.1 to 1 g; gravity specific PRE EMG is similar (1.5–1.75 g) or downregulated (0.5–0.75 g). **(B)** Based on this muscle stiffness regulation the GM muscle shows characteristic shortening in the three gravity conditions (0.5–0.75 g, 1 g and 1.5–1.75 g) before ground contact (GC). During 1 g the GM shows typically isometric behavior accompanied by neuromuscular enhancement. This optimized neuro-mechanical behavior (1 g) which favors efficient energy transfer is highlighted as bold black arrows. Altered neuro-mechanical behavior is observable during gravity conditions below or above 1 g. Diminished muscle activity during ECC seems to be interrelated to eccentric muscle stretch, followed by similar CON EMG and enhanced shortening to compensate ECC stiffness loss. Muscle behavior deviating from the model is highlighted as a bold dark gray arrow especially applying to 1.5–1.75 g.

The data reveal that gravity has a significant main effect on the complex motor control of ballistic movements modifying the EMG activity, muscle and MTU behavior, energy management, kinematics and performance indicators of the stretch-shortening cycle illustrated in Figure 3. To understand gravity-dependent adaptations of motor control we propose a mechanistic model, highlighted in Figure 4. This model illustrates the interaction between load-dependent stiffness regulation of the central nervous system and the contraction mode of the GM, determining the energy management.

### Kinetic Energy

**Figure 5:**
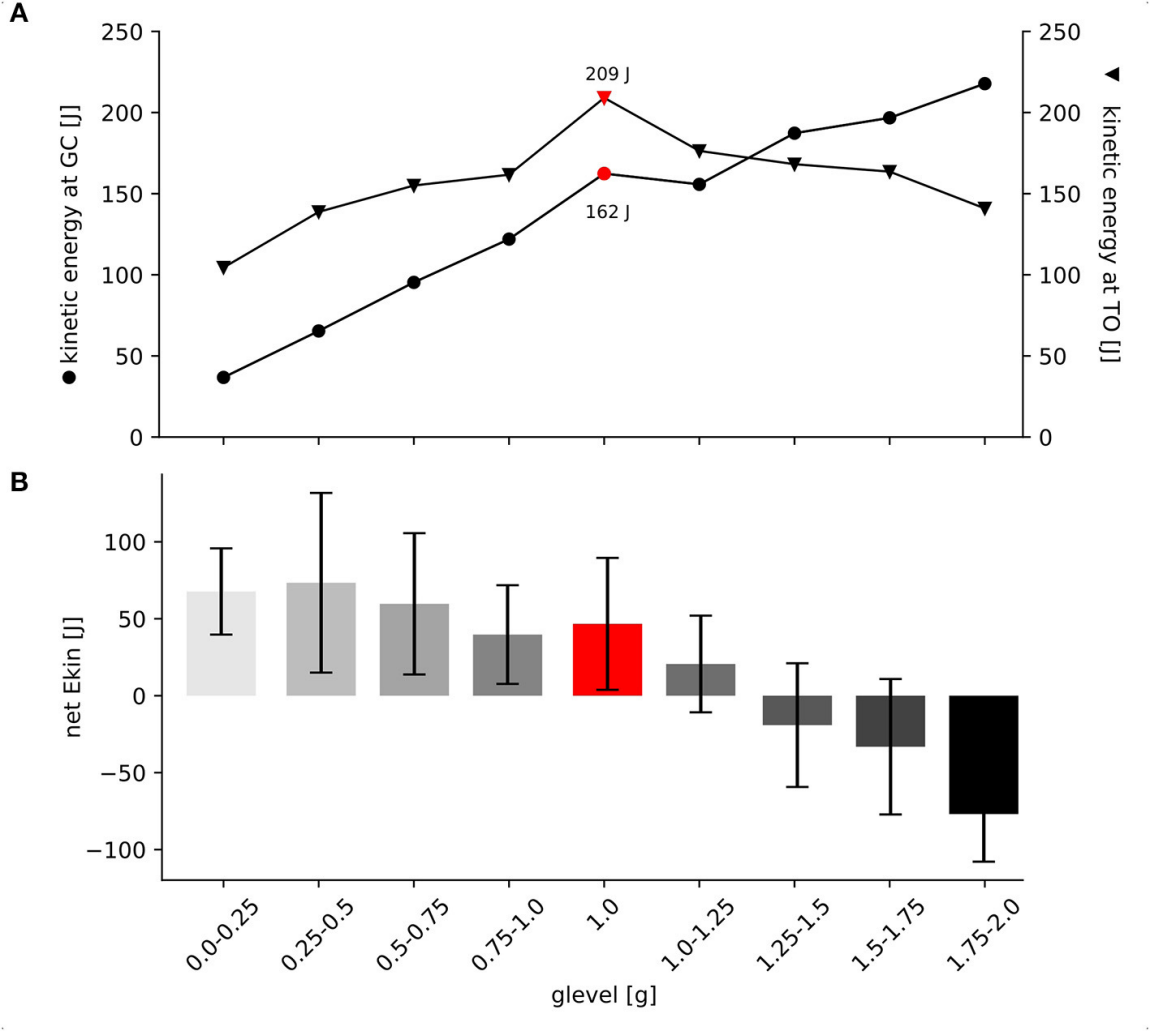
Net kinetic energy and kinetic energy at GC and take-off for all gravity conditions. **(A)** Grand means are illustrated for the kinetic Energy (E_kin_) at GC (black circles) and for E_kin_ at TO (black arrows), standard deviations are omitted for clarity. Data are presented as absolute values in Joule [J]. Up to 1 g, both time points show progressive increase as an effect of gravitational loading. Additional E_kin_ at take-off refers that the muscle-tendon complex may be able to efficiently store and release energy in parallel and serial elastic components during the stretch-shortening cycle up to 1.0–1.25 g. Above 1.25 g the significant interaction effect is visible as the two lines cross each other. In contrast to this, above 1.0–1.25 g less energy seems to be efficiently stored during the ECC, indicating a shift toward energy dissipation. **(B)** Grand means and standard deviations are shown for the net E_kin_ among all gravity conditions (0.1–2.0 g). Net E_kin_ was calculated as the difference between E_kin_ at ground contact (GC) and take-off (TO). Positive values indicate energy gain whereas negative values indicate energy loss during GC.

E_kin_ at GC progressively increases with increasing gravity ranging from 37 to 218 J (Supplementary Table 1). E_kin_ at take-off is highest in 1 g and diminishes gradually with decreasing and increasing gravity. Values range between 104 and 209 J (Supplementary Table 1). The mixed model ANOVA reveals a significant interaction of gravity level and E_kin_ at the two time points GC and take-off [*F*_(1,247.5)_ = 48.217, *p* < 0.001, η^2^ = 0.16] indicating either energy storage or dissipation of E_kin_ during the stretch-shortening cycle. The critical level was found to be 1.25 g (Figure 5).

### Performance Indicators and Stretch-Shortening Cycle Characteristics

**Figure 6:**
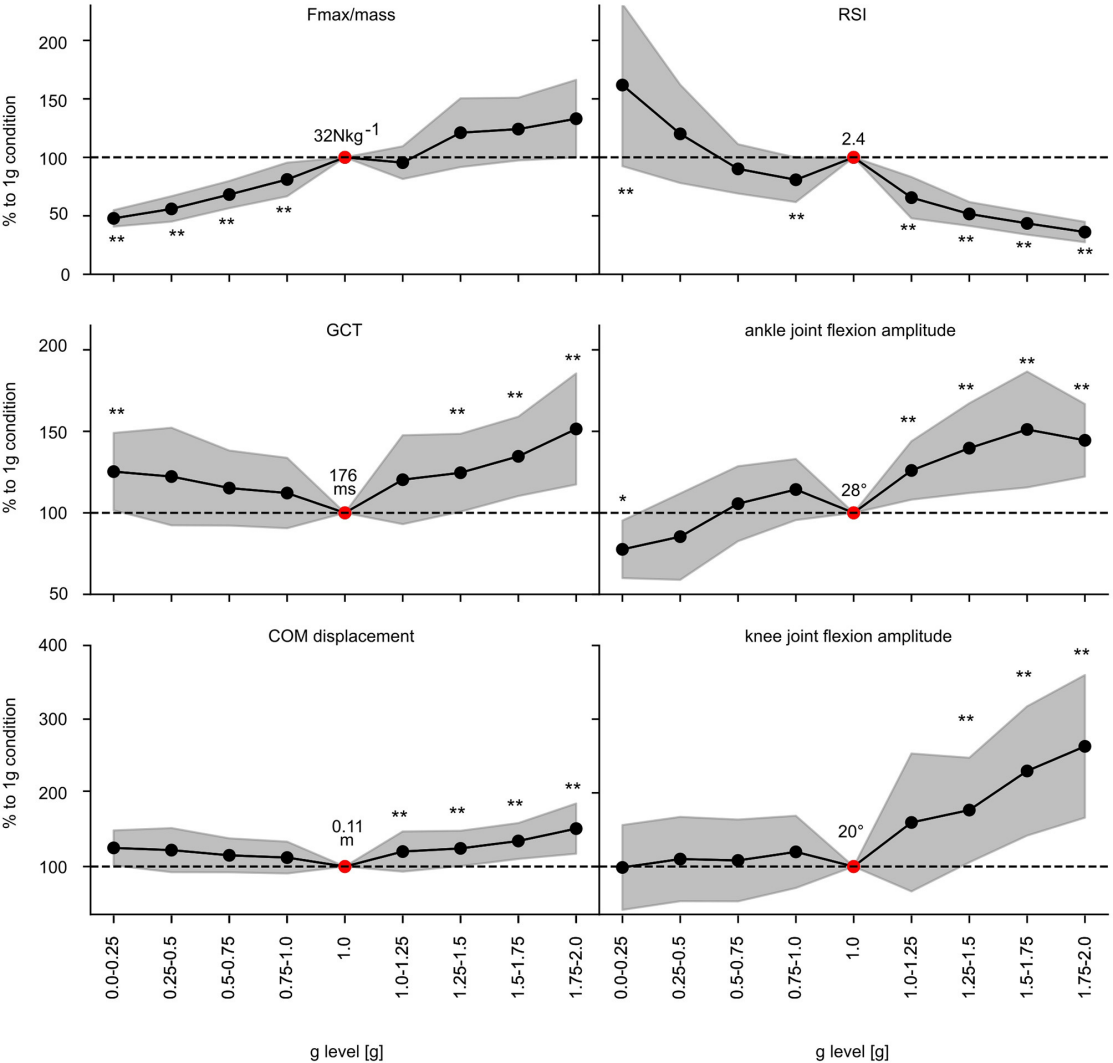
Effect of gravity on the most important parameters characterizing the performance within the stretch-shortening cycle. Grand means (*black marker*) and standard deviation (*gray area*) of the most important kinematic and performance indicator of the stretch-shortening cycle such as F_max_ normalized to body mass, reactive strength index (RSI), ground contact time (GCT), center of mass (COM) displacement and ankle and knee joint flexion amplitudes during ground contact for all subjects for the nine gravity clusters (0.0–0.25 g to 1.75–2.0 g). Data is normalized to the 1 g reference values (*dashed line* −−). Additionally, the 1 g condition is described by the absolute values (*red marker*). Gravity has a statistically significant influence on all of the displayed parameters (*p* < 0.05 mixed model ANOVA). Significant differences compared to the 1 g condition are marked with an asterisk (*p < 0.05, **p < 0.001).

Performance indicators show gravity-dependent modulations (Figure 6, Supplementary Table 1): F_max/mass_ progressively increases with increasing gravity. Thereby, significantly diminished values are reported for *F*_*max*/*mass*_ for all gravity levels below 1 g. GCT is shortest in 1 g and prolonged below and above 1 g. Accordingly, reactive strength index and JH are highest in 1 g while both are reduced below and above 1 g with two exceptions; in 0–0.25 g and 0.25–0.5 g the reactive strength index and JH significantly exceed the values of 1 g.

### EMG Activity

**Figure 7:**
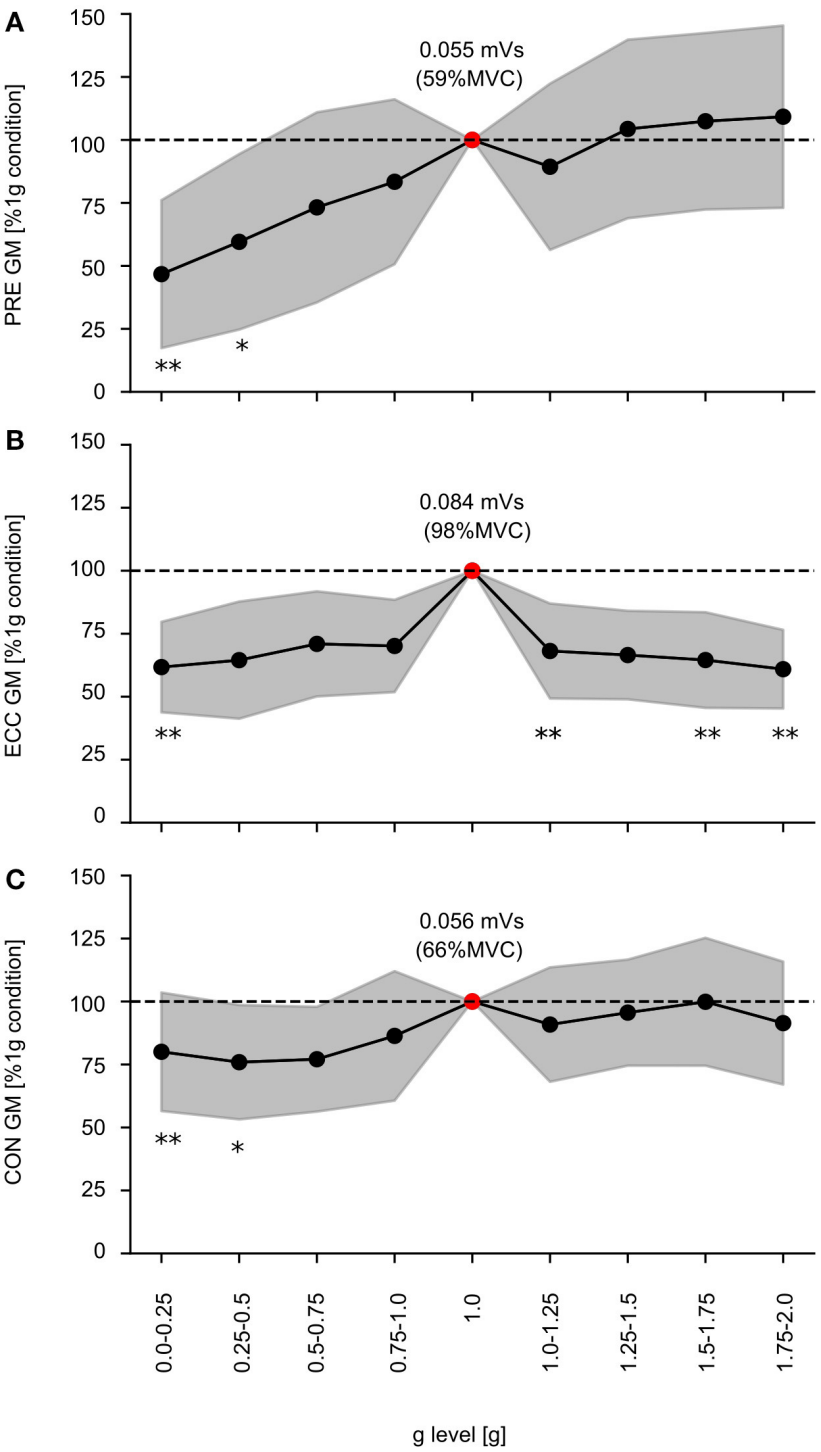
Electromyographic activity of the GM during the time intervals of the 1) preactivity (PRE), 2) eccentric (ECC), and 3) concentric phase (CON). Gravity has a statistically significant influence on EMG activity during every phase within the stretch-shortening cycle (*p* < 0.05 mixed model ANOVA), exemplarily depicted for the gastrocnemius medialis (GM) during PRE as well as the ECC and CON. Grand means (black marker) and standard deviation (gray area) for all subjects are illustrated for the nine gravity clusters (0.0–0.25 to 1.75–2.0 g). Data is normalized to the 1 g reference values (dashed line –). Additionally the 1 g condition is described by the absolute values (red marker). Significant differences compared to the 1 g condition are indicated with an asterisk (*p < 0.05, **p < 0.001).

The EMG activity of one exemplary subject is illustrated in Figure 3; grand means of EMG data for shank and thigh muscles are displayed in Supplementary Table 2. Gravity has a significant main effect on the EMG activity in PRE, ECC and CON for all recorded limb muscles involved in the ballistic movement (Supplementary Table 2). In PRE, the EMG activity progressively increases with increasing gravity ranging from 0.1 to 1 g. In ECC, the EMG activity rises from 0.1 to 1 g and subsequently declines from 1 to 2 g for all muscles despite TA. In CON, EMG activity of the antigravity muscles rises from 0.1 to 1 g and remains equal to 1 g beyond (Figure 7).

### Muscle-Tendon Mechanics

**Figure 8:**
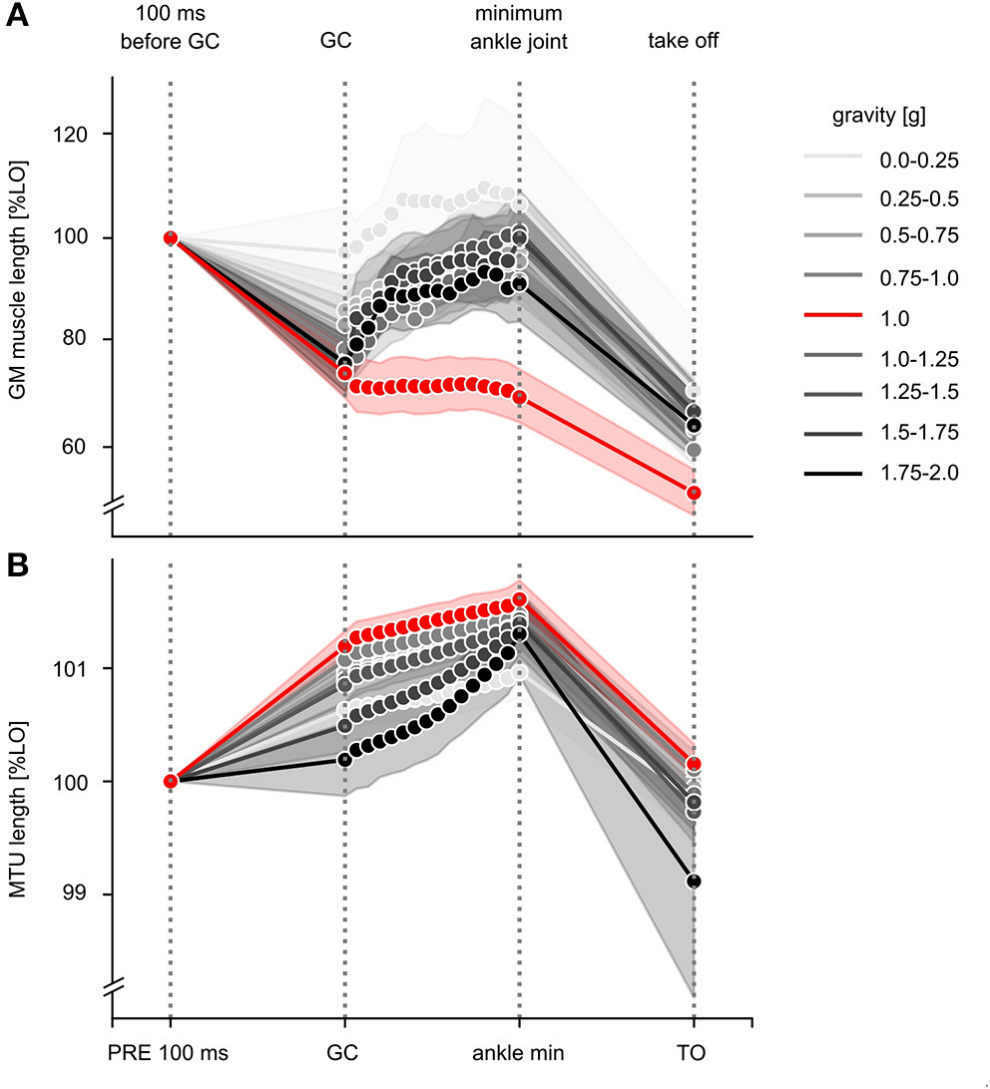
Effective length changes of the GM muscle and MTU during the stretch-shortening cycle for all gravity conditions. **(A)** Effective length changes of GM muscle and **(B)** muscle-tendon unit (MTU) during the stretch-shortening cycle movement are illustrated for the nine different gravity conditions ranging from 0.1 to 2 g. Data are presented as grand means and standard deviation and normalized to the corresponding gravity-specific length of 100 ms before GC (L0 PRE). Among all gravity levels, typical stretch-shortening cycle characteristics of the muscle-tendon mechanics are shown, such as muscle's shortening during PRE. During ECC, muscle behavior differs between the gravity conditions. Whereas, the reference jumps during 1 g show typical isometric behavior, especially above 1 g muscle lengthens eccentrically.

Gravity-induced changes in the effective GM muscle and MTU length are illustrated in Figure 8 for the time course of the ballistic movement. Grand means are displayed in Supplementary Table 3 differentiated by the MTU length (LMTU), effective muscle length (LM) and length of the tendinous tissue (LTT) for the periods PRE, ECC and CON (Figure 3). The mixed model ANOVA reveals a significant effect of gravity for the phases PRE, ECC and CON of the stretch-shortening cycle. In 1 g, effective GM muscle and MTU behavior is decoupled, in particular MTU exhibits a characteristic stretch-shortening cycle behavior during the GC phase: a shorting of the GM LM in PRE is followed by an isometric behavior for LM, lengthening of the LMTU in ECC and a subsequent shortening of the LMTU and LM in CON. Accordingly, the LTT lengthens in PRE and ECC. In gravity conditions below 1 g, the behavior of the LMTU and LTT for the phases PRE, ECC and CON is similar to 1 g: changes in LM indicate a slightly reduced shorting in PRE followed by modest elongation in the ECC movement. Accordingly, the LTT exhibits less lengthening in PRE and ECC.

Gravity conditions above 1 g differ to 1 g and cause a reduced lengthening of the LMTU in PRE concomitant with an elongation in ECC. LM shows a reduced shortening in PRE followed by a distinct elongation in ECC. The LTT prolonged in PRE and shortened in ECC.

### Ankle and Knee Joint Kinematics

The mixed model ANOVA reveals a significant main effect of gravity for the ankle and knee joint kinematics before and during ground contact (Supplementary Table 1). Ankle angles at initial GC are significantly increased from 0.5 until 2 g indicating an increased plantarflexion. Below 0.5 g the ankle angles at initial GC are similar compared to 1 g. Further, knee angles at initial GC are significantly increased above 1.25 g.

During ground contact, the results reveal significantly decreased maximal flexion amplitudes in the ankle joint in 0.0–0.25 g; whereas flexion amplitudes are increased above 1 g. Knee joint flexion amplitudes are also significantly increased (>1 g).

### Linear Mixed Models

Linear mixed model analysis uncovered that the event [GC vs. take-off], g level, PRE EMG, ECC EMG, and COM displacement [cm] are the most important parameters to predict the energy management during the GC phase. Our model proposes that the following equation can predict net E_kin_:

(6) $$\begin{array}{l} \text{net}\;{\text{E}}_{\text{kin}}=111.3\;\left(\text{event}\right)+\;61.0\;\left(\text{g}\;\text{level}\right)-79.8\;\left(\;\text{event}\;\text{x}\;\text{g}\;\text{level}\right)\\ \;+\;21.0\;\left(\text{ECC}\;\text{EMG}\right)+30.5\;\left(\text{PRE}\;\text{EMG}\right)+\;1.06\\ \;(\text{COM}\;\text{displ})-15.0\; \end{array}$$

The equation indicates that independent from gravity, energy can be gained during the GC phase by the given drop height used in the present study (CI 95% 97.6–125.1, se = 7.0, *p* < 0.001). Disregarding the event, E_kin_ would increase with increasing g level (CI 95% 43.2–78.8, se = 9.1, *p* < 0.001). Apparently, the interaction between gravity and the event is a crucial prerequisite to describe energy management during the GC phase. Findings support that above a critical limit, identified as 1.25 g, energy cannot be stored. In this context active absorption seems to be a logical interpretation emphasized by the diminished E_kin_ observed at take-off (CI 95% −93.0 to −66.6, se = 6.7, *p* < 0.001) (Figures 4, 5). Additionally, energy management during the GC phase is also interrelated to the amount of activity in PRE (CI 95% 1.3–59.7, se = 14.9, *p* = 0.041), ECC (CI 95% −3.615–45.6, se = 12.6, *p* = 0.095), and COM displacement (CI 95% −0.3–2.4, se = 0.7, *p* = 0.115). The mathematical relationship indicates that disregarding the event and gravity, increased PRE, ECC EMG and COM displacement would result in increased E_kin_.

Further, results show strong relation between F_max/mass_ and gravity (*F* = 670.53, se = 0.79, *p* < 0.001, CI 95% 18.92–22.02), indicating proportional increase in normalized F_max/mass_ by increasing gravity according to *F* = m^*^a. Additionally, the peak force is interrelated with the amount of muscle activity during ECC indicating that independent from gravity increase in ECC EMG results in increased F_max/mass_; by concerning both variables an increase would result in reduced peak force:

(7) $$\begin{array}{l} {\text{F}}_{\text{max}/\text{mass}}=20.47\;\left(\text{g}\;\text{level}\right)+11.1\;\left(\text{ECC}\;\text{EMG}\right)-7.78\\ \;(\text{g}\;\text{level}\;\text{x}\;\text{ECC}\;\text{EMG})+\;6.12\; \end{array}$$

## Discussion

The current study provides new knowledge about the influence of gravity and thus acceleration on ballistic movement dynamics. We demonstrated that the central nervous system seems to anticipate varying acceleration conditions and regulates skeletal muscle contraction to achieve a reactive movement pattern with an appropriate energy management. However, neuro-mechanical coupling appears to be optimized just in 1 g. Below 1 g muscle activity is suboptimal, leading to substantial length changes in the muscle and thus compromising storage and release of elastic energy in the tendon. Above 1 g the mode of energy management during the GC phase shifts from energy storage to energy dissipation due to diminished muscle activity and forceful longitudinal muscle lengthening. As a consequence, it appears that the load dependency of the muscle-tendon complex requires specific motor control strategies to achieve an optimized neuro-mechanical coupling.

### Optimized Neuro-Mechanical Coupling in 1 g

Jumping in 1 g conditions turned out to reflect optimized neuro-mechanical coupling compared to all other gravity conditions. An appropriate pre-activity and intense muscle activity after GC ensured a quasi-isometric muscle contraction during the eccentric phase of the GC phase. The amount of muscle activity before GC is pre-programmed on the prediction of the instant and velocity of muscle stretch before initial touch-down partially based on vestibular input (Melvill Jones and Watt, 1971; McDonagh and Duncan, 2002). Additionally, superimposed stretch reflexes are able to further support muscle stiffness during the GC phase allowing high stiffness of the muscle fibers (Gollhofer et al., 1992; Komi and Gollhofer, 1997) crucial for utilizing the spring-like properties of the muscle-tendon complex (Gollhofer and Kyröläinen, 1991; Komi and Gollhofer, 1997; Arampatzis et al., 2004). The superimposed reflex activity has been suggested to contribute to high short range elastic stiffness (SRES), which is known to prevent active cross-bridges from yielding (Rack and Westbury, 1974). Alternatively it has been shown that activated muscle fibers are further able to form links between titin and actin filaments (N2A mechanism). Through calcium dependent binding of the N2A region of titin to the actin filament, stiffness of the contractile system is increased (Lindstedt and Nishikawa, 2017). We argue that under such conditions, lengthening of the MTU allows optimal stress/strain interaction and thus energy storage in the tendinous tissue.

### Neuro-Mechanical Coupling Below 1 g

The typical sequential activity pattern in the stretch-shortening cycle with PRE, ECC and CON was observed among all gravity conditions below 1 g. The results demonstrate a systematic increase in pre-activity from 0.1 to 1 g; however both pre-activity and ECC muscle activity were diminished compared to 1 g. The results indicate that gravity, and the resulting kinetic energy at GC, seems to be anticipated before touch-down. It has been shown that muscle activity before GC is sensitive to various loading conditions; thus, pre-activity levels increase with increasing stretch loads (Gollhofer and Kyröläinen, 1991; Avela et al., 1996; Ishikawa et al., 2003). Furthermore, Gambelli et al. (2016) demonstrated that subjects modify their motor commands according to the amount of energy gained during the fall. Taking advantage of the anticipatory sensory capacity (McDonagh and Duncan, 2002), subjects likely aimed at smoothening the movement during GC (Ritzmann et al., 2016) making use of the pre landing muscle activity regulated by higher brain structures (Taube et al., 2012). Ritzmann et al. (2016) found that the muscle activity before GC was tailored to the corresponding gravitational condition and were in accordance with force profiles reflected by F_max/mass_. Hence, our results match previously reported interrelations between force profiles (reduced force-generating capacity) and muscle activity before and during ECC with strong coincidence with the recoil properties of the muscle-tendon complex (Komi, 2000; Ritzmann et al., 2016). In particular, during our experiments reduced muscle activity before and during ECC was accompanied by longitudinal muscle lengthening. Therefore, we conclude that muscle stiffness regulation before and during ECC are suboptimal to fully benefit from the spring-like behavior in response to the eccentric load induced by reduced dynamics. In contrast to preliminary literature our results do not confirm the assumption of increased take-off velocity in reduced external load conditions such as in reduced gravity (Cavagna et al., 1972). Those discrepancies can be explained by differences in the experimental setups; in contrast to our study Cavagna et al. (1972) have performed different kind of vertical jumps, further the authors have calculated the COM velocity. Nevertheless, the compromised spring-like behavior seems to be sufficient to achieve a ballistic mode of jumping in low gravity conditions. Reduced neuromuscular activity alters SRES of cross bridges. Gollhofer and Kyröläinen (1991) hypothesized that low stretch loads might be mechanically compensated by smaller joint flexion amplitudes of the knee and ankle, in order to maintain sufficient loading rates in the ECC phase. This mechanical compensation is evident in the lowest gravity condition (0.0–0.25 g). Above 0.25 g it rather seems possible that the reduced take-off velocity is compensated by similar joint flexion amplitudes (Cavagna et al., 1972). Assuming that the work performed by the muscle is similar among a wide range of gravity profiles, the force-velocity relationship would imply that the increased net kinetic energy might be a result of the reduced external requirements and the increased shortening velocities of the muscles upon the maximal speed of shortening (Cavagna et al., 1972).

### Neuro-Mechanical Coupling Above 1 g

Above 1 g, the pre-activity is comparable in magnitude compared to 1 g. A high pre-activity as reached during maximal voluntary contraction (Supplementary Table 2) is important to provide the MTU with appropriate stiffness to resist the increasing impact loads and to meet the criteria of an efficient stretch-shortening cycle (Komi and Gollhofer, 1997). From kinematic perspective the increased plantarflexion and knee extension above 1.25 g could be an important task-specific strategy to ensure appropriate stiffness during ballistic movements in contrast to controlled landings (Gambelli et al., 2015). Increased ankle and knee angles at GC could also represent an important modification in order to increase the dampening distance which has already been discussed in the context of landings (Minetti et al., 1998). Moreover, muscle activity in the eccentric phase is extensively reduced. Applying increased drop heights has been approved that pre-activity, reflex activity, the recoil properties of the MTU and performance related parameters such as jump height show a progressive increase as a function of stretch load (Asmussen and Bonde-Petersen, 1974; Schmidtbleicher and Gollhofer, 1982; Ishikawa et al., 2003). However, various experiments determined an individual physiological breaking point beyond which the maximal jumping performance cannot be further enhanced (Komi and Bosco, 1978). Beyond this individual optimal stretch load, performance relevant parameters are modified. Taube et al. (2012) postulated that reduction in reflex contribution during ECC in response to increased drop heights progressively changes the mechanical properties of the muscle from high and efficient stiffness to a more compliant state. Assuming that superimposed stretch reflexes contribute to high eccentric stiffness (Zuur et al., 2010), appropriate SRES levels might be accompanied. Jumping with individually extremely high impact loads showed that in disproportionate high drop heights, the H-reflexes are diminished, following the idea of a safety strategy (Komi and Gollhofer, 1997; Leukel et al., 2008a,b). It has been argued that inhibitory mechanisms are involved in controlling the reflex activity in order to protect musculotendinous structures from damage (Leukel et al., 2008a,b). Loading in gravity conditions above 1 g are probably linked to conditions with exceeding falling heights. In such conditions it seems logically that muscle stiffness before and during ECC is inadequate to secure high SRES. As a consequence, the muscle is forcefully lengthened, thus compromising muscle-tendon interaction (Sousa et al., 2007). Functionally, when the muscle and tendon “safety factors” are exceeded, muscle-tendon mechanics shifts toward a more compliant system which might be associated with energy dissipation (Werkhausen et al., 2017). These factors are basically defined by “the ratio of failure stress to peak functional, or operating, stress” (Biewener, 2005, p. 1665). One of the most critical structure's safety factors is the amount of strain energy which can “[…] be absorbed during a loading cycle relative to the work of fracture of a material (e.g., bone or tendon) […]” (Biewener, 2005, p. 1666). In general, stretch load appears to be individually tolerable and limited by the muscle-tendon safety factors (Biewener, 2005). Biomechanically, these consequences are reflected by increased joint flexion amplitudes of the lower limb. It can be assumed that under high strain conditions, the observed alterations of joint kinematics are based on the reduced muscle activity after GC. On the other hand, altered joint kinematics such as increased joint flexion has also been reported as an active response to increased joint loads (Peng, 2011; Lesinski et al., 2018). Increased flexion is described as an efficient knee motion strategy to compensate high joint loads. Mechanically, these adaptations result in loss of reactive performance (Gollhofer and Kyröläinen, 1991; Avela et al., 1996; Kramer et al., 2012; Helm et al., 2019, 2020).

### Modeling and Associated Mechanisms

During the last decades, the potential mechanisms associated with load-dependent modulations of the stretch-shortening cycle have been intensively discussed. Stiffness regulation on the neuromuscular level, including phase-specific modulation of muscle activity, is important to benefit from the spring-like properties of the MTU (Gollhofer et al., 1992; Avela et al., 1994).

However, both mechanisms cannot be considered independently as they both interact functionally. From our understanding, the neuro-mechanical coupling is the driving mechanism determining energy storage and release and thus power enhancement during a wide range of gravity profiles (0.1–2 g). Based on our experimental data, we calculated LMM to detect the contributing key determinants of the net energy outcome function during the stretch-shortening cycle. The mechanistic model is illustrated in Figure 4. Load specific neuro-mechanical coupling can be modulated as a function of gravity related to the economy of movement and performance enhancement during ballistic movements. In particular, to keep the modulation formula as comprehensive as possible we predicted the net kinetic energy by including the gravitational acceleration, pre-activity, eccentric activity and the center of mass displacement into the model. The independent variables were selected based on previously mentioned arguments.

As we intended to predict net E_kin_ as the key variable for energy management to obtain a comprehensive understanding of the net energy output, it is not surprising that the event [ground contact vs. take-off] is the most important parameter. The interaction term between event and g level, is the second important determinant to describe net E_kin_ during stretch-shortening cycle. By considering both factors independently (event and g level), we can assume that the given drop height of 25 cm, was high enough to enable energy storage in the MTU. This energy gain and storage is highlighted by the positive signs of event and g level. This allows the system to accelerate the COM at TO, reflected by systematically increased jump heights and performance enhancement. Such a beneficial energy management can be observed and verified by our experimental data from 0.1 to 1 g. As a result of this, the neuromuscular configuration included in the formula as PRE and ECC EMG are important factors allowing to meet the criteria of an efficient stretch-shortening cycle established by Komi and Gollhofer (1997). In their understanding “an effective stretch-shortening cycle requires three fundamental conditions: an early high-magnitude pre-activity of the muscle(s) before the eccentric phase, a short and fast eccentric phase, and immediate transition (short delay) between stretch (eccentric) and shortening (concentric phase)” (Komi and Gollhofer, 1997, p. 451). Ritzmann et al. (2016) have presented the first data of ballistic movements in reduced gravity conditions. Interestingly, significant correlations between the muscle activity during the pre-activity phase and rate of force development (*r* = 0.89^*^, *p* < 0.01) and the muscle activity during the late latency response and rate of force development (*r* = 0.91^*^, *p* < 0.01) were detected for the GM. Authors postulated that in such conditions, GM is primarily controlled by supra-spinal areas (Mrachacz-Kersting et al., 2006). This interrelation further emphasizes that systematic increase of muscle activity before and during the GC phase changes the longitudinal muscle behavior from suboptimal lengthening to an isometric behavior. Anchoring of the muscle during ECC, as seen in the 1 g condition allows the system to increase tendinous tissue stretch. In total, the amount of net E_kin_ can be enhanced from 0.1 to 1 g. Again the optimal neuro-mechanical coupling is verified by LMM for the 1 g condition allowing beneficial net E_kin_ and maximized performance. In contrast to these systematic interrelations we have detected a breaking point also verified by the LMM and manifested at 1.25 g. In particular, the numerical differences between E_kin_ at GC and take-off in gravity levels beyond 1.25 g highlight ineffective energy management. The functional importance of the MTU of the triceps surae during ECC is used differently. It is adapted to dampen the movement associated with energy dissipation rather than energy storage (Lindstedt et al., 2001, 2002; Werkhausen et al., 2017) to protect muscles from damage associated with rapid active stretch and release (Griffiths, 1991; Reeves and Narici, 2003; Konow et al., 2012). Overall we can highlight that deviations from the optimized neuro-mechanical coupling established during the 1 g condition induce disproportionate alterations in the neuro-mechanical coupling.

### Limitations

**Figure 9:**
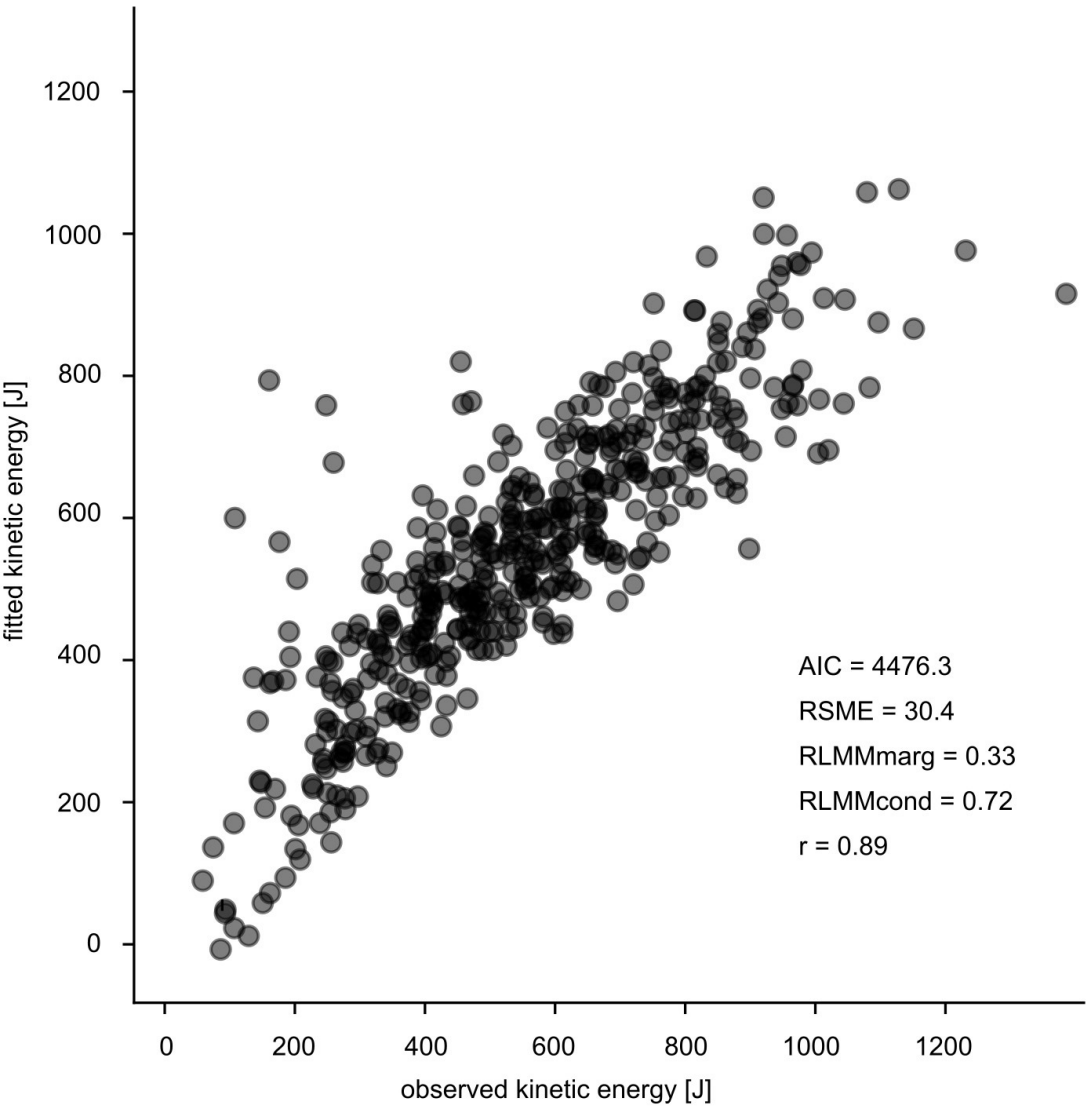
Goodness of fit of the LMM with the corresponding values. The observed values (E_kin_ [J]) are illustrated on the x-axis and the fitted values (E_kin_ [J]) are presented on the y-axis.

For a conclusive statement, it is crucial to consider the limitations of the study. Three aspects are of substantial importance: (i) For this unique experimental setup (parabolic flights), the reported sample size consisting of 17 subjects is appropriate (Gambelli et al., 2016; Ritzmann et al., 2016); however, from a statistical point of view the sample size might appear to be rather small. (ii) In order to account for the hierarchical structure of the experimental dataset, LMM was chosen although linear regression analyses are more familiar and intuitive by reporting typical *R*^2^. In our case, *R*^2^ has been approximated and thus the current outcomes are not directly comparable with the results of similar studies. However, LMM delivers good estimates of the kinetic energy and energy management (AIC = 4476.33, RSME = 30.4, Figure 9). (iii) Measurement technology, methodology and the data processing have restrictions such as the limited sampling frequency of kinematics, the approximation with the use of the pelvis marker for a COM estimate, and the subsequent errors on the estimation of the kinetic energy. Because our focus was primarily set on the muscle model of the plantarflexors the timely division between eccentric and concentric phase based on the ankle angle is an appropriate procedure well-established in literature (Kubo et al., 2007a,b; Hoffrén et al., 2011). From global perspective defining the transition point based on the COM trajectory would have been an appropriate alternative. Furthermore future studies should address determinants such as the spring mass model (Blickhan, 1989; Cavagna et al., 2005) to get a more comprehensive integrative understanding of the mechanical function during bouncing gait.

## Conclusion and Functional Relevance

For the first time muscle activity profiles of lower limb muscles concerning energy management were evaluated during reactive drop jumps in a broad spectrum of artificial gravity (parabolic flight) conditions ranging from 0.1 to 2 g. The present findings highlight that optimized neuro-mechanical coupling exclusively observed during the 1 g condition cannot be reproduced in altered gravity conditions to the same extent. Optimal control is argued to be characterized by high muscle activity before and during GC, enabling isometric muscle behavior. As a consequence, the energy at take-off is optimized and highest in 1 g. Scaling acceleration below 1 g, suboptimal muscle activity induces substantial length changes in the muscular tissue and thus compromising effective elastic energy transfer in the tendon. It is intuitive that with progressively reduced acceleration the needs for load compensation are diminishing, inducing systematic downregulation of muscle activity compromising all biomechanical constraints necessary to perform short ground contact times as compared to 1 g. Above 1 g the mode of energy management shifts from energy storage to energy dissipation due to diminished muscle activity and forceful longitudinal muscle lengthening. In our functional approach we explained with our model the kinetic energy outcome by a linear mixed model encountering the differences between E_kin_ at GC and E_kin_ at TO as the net criteria of energy turnover. This linear mixed model revealed high validity (*r* = 0.89).

## Data Availability Statement

The raw data supporting the conclusions of this article will be made available by the authors, without undue reservation.

## Ethics Statement

The studies involving human participants were reviewed and approved by Ethics Committee of the University of Freiburg. The patients/participants provided their written informed consent to participate in this study.

## Author Contributions

JW, RR, KF, MH, EM, BS, KA, AG, and MN conceived, designed the research, and approved the final version of the manuscript. JW, RR, KF, MH, EM, BS, KA, and MN performed the experiments. JW, KF, RR, and EM analyzed the data. JW, KF, RR, AG, and MN interpreted the results of the experiments. JW prepared the figures. JW and RR drafted the manuscript. JW, RR, KF, EM, AG, and MN edited and revised the manuscript. All authors agree to be accountable for all aspects of the work in ensuring that questions related to the accuracy or integrity of any part of the work are appropriately investigated and resolved. All persons designated as authors qualify for authorship, and all those who qualify for authorship are listed.

## Conflict of Interest

The authors declare that the research was conducted in the absence of any commercial or financial relationships that could be construed as a potential conflict of interest.
