# Supplementary material for: The Anticipation of Gravity in Human Ballistic Movement
Source: Front Physiol. 2021 Mar 17;12:614060. doi: 10.3389/fphys.2021.614060 (PMC8010298; doi:10.3389/fphys.2021.614060)
Supplement: Supplementary file 1 [file Data_Sheet_1.pdf]

Supplementary Material

1 Tables

Table S1. Influence of gravity on ground reaction forces (GRF), Stretch-shortening cycle characteristics, kinetic energy, and joint kinematics

	**Gravity level**									**Mixed model ANOVA (F, p, n^2)**
	**0.0-0.25**	**0.25-0.5**	**0.5-0.75**	**0.75-1.0**	***1.0***	**1.0-1.25**	**1.25-1.5**	**1.5-1.75**	**1.75-2.0**	**fixed effect gravity**
**$F_{max/mass}$ [Nkg^{-1}]**	15 ±2**	18 ±3**	22 ±3**	26 ±5**	*32 ±5*	29 ±4	37 ±10	37 ±6	39 ±9	**F(1, 195.3) = 36.540 p < 0.001, n^2 = 0.16**
**GCT [ms]**	225 ±43**	213 ±49	201 ±40	198 ±40	*176 ±26*	207 ±40	218 ±46**	236 ±48**	266 ±69**	**F(1, 191.1) = 27.851, p < 0.001, n^2 = 0.13**
**jump height [m]**	0.82 ±0.26**	0.59 ±0.18**	0.42 ±0.09	0.37 ±0.08	*0.42 ±0.08*	0.32 ±0.07**	0.26 ± 0.05**	0.24 ±0.04**	0.22 ±0.03**	**F(1, 218.0) = 81.514, p < 0.001, n^2 = 0.27**
**RSI [ratio]**	3.7 ±0.9**	2.8 ±0.9	2.2 ±0.5	1.9 ±0.5**	*2.4 ±0.6*	1.6 ±0.5**	1.2 ±0.3**	1.1 ±0.3**	0.9 ±0.2**	**F(1, 202.1) = 66.455, p < 0.001, n^2 = 0.25**
**COM displacement [m]**	0.09 ±0.03	0.11 ±0.03	0.12 ±0.03	0.13 ±0.02	*0.11 ±0.03*	0.14 ±0.03**	0.16 ±0.03**	0.18 ±0.03**	0.20 ±0.03**	**F(1, 193.6) = 55.798 p < 0.001, n^2 = 0.22**
**E_{kin} GC [joule]**	37 ±7**	65 ±20**	95 ±22**	122 ±31**	*162 ±45*	156 ±37	187 ±37**	197 ±44**	218 ±38**	**F(1, 197.6) = 71.482, p < 0.001, n^2 = 0.27**
**E_{kin} TO [joule]**	104 ±29**	139 ±61**	155 ±59**	162 ±54**	*209 ±65*	176 ±56	168 ±57**	164 ±63**	141 ±50**	**F(1, 207.2) = 11.63, p < 0.001, n^2 = 0.05**
**ankle angle GC [°]**	137 ±8	137 ±8	139 ±7**	141 ±8**	*132 ±8*	142 ±7**	143 ±6**	143 ±6**	142 ±5**	**F(1, 146.9) = 29.334, p < 0.001, n^2 = 0.17**
**knee angle GC [°]**	154 ±6	153 ±7	153 ±8	154 ±7	*151 ±8*	155 ±5	156 ±5*	159 ±6**	160 ±5**	**F(1, 166.2) = 9.787, p < 0.001, n^2 = 0.06**
**ankle flexion amplitude [°]**	23 ±6*	24 ±8	29 ±7	32 ±6	*28 ±7*	35 ±6**	37 ±5**	39 ±6**	39 ±6**	**F(1, 142.4) = 44.182, p < 0.001, n^2 = 0.24**
**knee flexion amplitude [°]**	18 ±9	20 ±9	20 ±9	22 ±7	*20 ±8*	26 ±8	32 ±9**	39 ±7**	46 ±7**	**F(1, 171.3) = 51.462, p < 0.001, n^2 = 0.23**

Changes in $F_{max/mass,}$ ground contact time (GCT), jump height, reactive strength index, vertical center of mass (COM) displacement during the GC phase, kinetic energy at GC (E_{kin} GC) and take-off (E_{kin} TO), ankle and knee joint kinematics at initial GC and joint flexion amplitudes during the GC phase are presented as mean values ± standard deviation (SD). Bolt p and F values indicate a significant main effect of gravity. Effect sizes are given by η^2. Significant differences compared to the 1g condition are marked with an asterisk (* for $p < 0.05$; ** for $p < 0.001$).

Table S2. Gravity-related differences of electromyographic activity

	**Gravity level**									**Mixed model ANOVA (F, p, n^2)**
	**0.0-0.25**	**0.25-0.5**	**0.5-0.75**	**0.75-1.0**	**1.0**	**1.0-1.25**	**1.25-1.5**	**1.5-1.75**	**1.75-2.0**	**fixed effect gravity**
**PRE**										
**SOL**	43 ±24*	59 ±41*	66 ±26	83 ±36	*24 ±17*	95 ±38	119 ±48	121 ±55	122 ±57	**$F(1, 363.3) = 12.841$, $p <0.001$, $n^2 = 0.03$**
**GM**	47 ±29**	60 ±35*	73 ±38	83 ±33	*59 ±26*	89 ±33	104 ±35	107 ±35	109 ±36	**$F(1, 390.3) = 16.944$, $p < 0.001$, $n^2 = 0.04$**
**GL**	48 ±23**	53 ±30**	73 ±43*	84 ±41	*48* ±12	97 ±42	115 ±51	118 ±50	125 ±65	**$F(1, 172.6) = 15{,}271$, $p <0.001$, $n^2 = 0.08$**
**TA**	45 ±30**	60 ±32**	70 ±25**	87 ±25	*28 ±15*	94 ±27	114 ±37	118 ±40	117 ±40	**$F(1, 154.1) = 10{,}923$, $p < 0.001$, $n^2 = 0.07$**
**VM**	*55 ±38	71 ±59	87 ±52	100 ±43	*19 ±9*	130 ±75	140 ±93	119 ±58	124 ±64	**$F(1, 158.2) = 4.932$, $p < 0.001$, $n^2 = 0.03$**
**RF**	51 ±35**	63 ±40*	81 ±35*	110 ±52	*13 ±7*	140 ±75	158 ±81	153 ±78	156 ±91	**$F(1, 181.3) = 10.877$, $p < 0.001$, $n^2 = 0.06$**
**BF**	58 ±30**	60 ±34*	69 ±40*	82 ±54	*22 ±10*	93 ±76	104 ±65	97 ±55	101 ±67	**$F(1, 155.3) = 4.676$, $p < 0.001$, $n^2 = 0.03$**
**ECC**										
**SOL**	58 ±19**	61 ±21**	69 ±20**	71 ±20**	*80 ±41*	71 ±21	71 ±20**	75 ±21**	72 ±18**	**$F(1, 154.1) = 11.531$, $p < 0.001$, $n^2 = 0.07$**
**GM**	62 ±18**	65 ±23	71 ±21	70 ±18	*98 ±53*	68 ±19**	67 ±18	65 ±19**	61 ±16**	**$F(1, 432.3) = 6.227$, $p <0.001$, $n^2 = 0.01$**
**GL**	68 ±25**	71 ±21**	75 ±21	77 ±17**	*87* ±34	76 ±24	79 ±30	75 ±20*	70 ±17**	**$F(1, 195.7) = 9.987$ $p < 0.001$, $n^2 = 0.05$**
**TA**	100 ±23	104 ±43	103 ±33	122 ±71	*20 ±10*	124 ±61	119 ±42	133 ±42	143 ±62	**$F(1, 184.5) = 2.952$, $p = 0.004$, $n^2 = 0.02$**
**VM**	49 ±11**	58 ±15**	66 ±17**	73 ±21**	*102 ±45*	82 ±24	88 ±19	89 ±17	92 ±19	**$F(1, 138.5) = 8.784$, $p < 0.001$, $n^2 = 0.06$**
**RF**	39 ±14**	47 ±17	59 ±16	68 ±13	*78 ±37*	83 ±23	88 ±27**	87 ±12**	87 ±13**	**$F(1, 131.1) = 14.918$, $p < 0.001$, $n^2 = 0.102$**

**BF**	49<br>±13**	60<br>±29**	65<br>±36**	58<br>±21**	*39*<br>*±19*	62<br>±22	60<br>±28**	71<br>±32	66<br>±32*	**F(1, 177.8) = 10.647,**<br>**p < 0.001, n^2 = 0.06**
**CON**										
**SOL**	83<br>±32	78<br>±23	77<br>±24*	88<br>±28*	*57*<br>*±33*	92<br>±28	94<br>±20	102<br>±24	101<br>±33	**F(1, 166.9) = 3.201,**<br>**p = 0.002, n^2 = 0.02**
**GM**	80<br>±24*	76<br>±23	77<br>±21**	86<br>±26	*66*<br>*±46*	91<br>±23	96<br>±21	100<br>±25	91<br>±24	**F(1, 198.6) = 3.137,**<br>**p = 0.002, n^2 = 0.02**
**GL**	77<br>±21**	82<br>±23	77<br>±19*	90<br>±30	*65*<br>*±32*	90<br>±21	98<br>±25	103<br>±28	94<br>±26	**F(1, 267.9) = 2.192,**<br>**p < 0.001, n^2 = 0.01**
**TA**	113<br>±32	100<br>±51	107<br>±52	117<br>±47	*18*<br>*±9*	112<br>±45	125<br>±66	127<br>±56	132<br>±56	F(1, 150.9) = 1.817,<br>p = 0.078, n^2 = 0.01
**VM**	61<br>±35	61<br>±26**	58<br>±22**	71<br>±33*	*75*<br>*±48*	83<br>±25	93<br>±32	85<br>±26	83<br>±31	**F(1, 419.1) = 7.233,**<br>**p < 0.001, n^2 = 0.02**
**RF**	72<br>±49	68<br>±22**	69<br>±26**	78<br>±31	*50*<br>*±28*	86<br>±22	96<br>±32	100<br>±35	99<br>±40	**F(1, 162.0) = 4.839,**<br>**p < 0.001, n^2 = 0.03**
**BF**	59<br>±21**	61<br>±28**	61<br>±19**	68<br>±20**	*40*<br>*±19*	74<br>±18	85<br>±26	90<br>±25	79<br>±25	**F(1, 210.4) = 10.261,**<br>**p < 0.001, n^2 = 0.05**

Changes in electromyographic (EMG) activity in shank and thigh muscles [normalised to iEMG during maximal voluntary contraction and time normalised] regarding gravity variation in the relevant phases: PRE (100 ms before GC until touch-down), ECC (touch-down until minimal ankle joint flexion) and CON (min ankle joint flexion until take-off). For *1g*, absolute values are represented as mean values, and SD normalised to MVC iEMG. For gravity conditions below and above 1g, data is normalised to the 1g values. Relative values are expressed as %. Bolt p and F values indicate a significant main effect of gravity. Effect sizes are given by η^2. Significant results are marked with an asterisk (* for $p < 0.05$; ** for $p < 0.001$).

Table S3. Gravity-related length changes in the muscle, muscle-tendon length and tendinous tissue. The terms PRE, ECC and CON refer to lengthening or shortening of the entire MTU before (PRE) and during the GCT (ECC and CON) according to the EMG time intervals.

	**Gravity level**									**Mixed model ANOVA (F, p, n²)**
	**0.0-0.25**	**0.25-0.5**	**0.5-0.75**	**0.75-1.0**	***1.0***	**1.0-1.25**	**1.25-1.5**	**1.5-1.75**	**1.75-2.0**	**fixed effect gravity**
**LM**										
**PRE**	-0.4 ±2.2 **	-5.1 ±4.0 *	-6.1 ±4.8 *	-7.0 ±4.1	*-11.7 ±6.9*	-7.9 ±3.6	-8.0 ±4.3	-8.3 ±4.0	-7.4 ±3.5 *	**F(1, 148.5) = 5.296, p < 0.001, n² = 0.03**
**ECC**	+3.2 ±4.6**	+2.2 ±4.5**	+3.2 ±3.4**	+3.5 ±4.9	*-1.8 ±3.0*	+6.6 ±3.3**	+7.2 ±3.1**	+7.0 ±5.3**	+4.4 ±4.2**	**F(1, 220.8) = 9.720, p < 0.001, n² = 0.04**
**CON**	-9.2 ±1.3	-10.8 ±4.5	-8.5 ±2.9	-9.1 ±4.7	*-6.1 ±3.7*	-9.7 ±2.4	-9.3 ±2.2	-9.0 ±4.3	-7.8 ±4.0	F(1, 190.9) = 0.898, p = 0.532, n² = 0.01
**LMTU**										
**PRE**	2.6 ±0.9 *	3.5 ±0.9	3.6 ±1.2	3.7 ±1.2	*4.4 ±1.4*	3.6 ±1.6	2.9 ±1.7	1.8 ±2.0**	0.5 ±2.9**	**F(1, 206.5) = 8.435, p <0.001, n² = 0.04**
**ECC**	1.6 ±1.1	1.8 ±1.6	1.4 ±1.3	1.6 ±1.0	*1.5 ±1.0*	1.7 ±1.2	2.1 ±1.4	3.4 ±2.3*	4.3 ±2.7**	**F(1, 203.0) = 6.171, p < 0.001, n² = 0.03**
**CON**	-4.4 ±1.8	-5.1 ±1.9	-5.2 ±1.8	-5.0 ±1.6	*-5.3 ±1.5*	-5.6 ±1.5	-6.1 ±1.8	-5.7 ±1.8	-7.7 ±4.1	**F(1, 250.7) = 2.367, p = 0.018, n² = 0.01**
**LTT**										
**PRE**	2.9 ±2.1**	8.6 ±4.4	9.7 ±5.7*	10.7 ±4.9	*16.1 ±7.6*	11.5 ±4.4	11.0 ±5.2	10.1 ±5.6*	7.9 ±4.8**	**F(1, 196.8) = 6.466, p < 0.001, n² = 0.03**
**ECC**	-1.6 ±3.9**	-0.6 ±4.8	-1.8 ±4.0**	-2.0 ±5.0**	*3.2 ±3.3*	-4.9 ±3.2**	-5.1 ± 3.0**	-3.6 ±5.0**	0.0 ±3.9	**F(1, 231.8) = 7,313, p < 0.001, n² = 0.03**
**CON**	4.8 ±1.8	6.4 ±5.7	3.3 ±3.4	4.1 ±5.2	*1.4 ±2.8*	4.1 ±2.0	3.2 ±3.3	3.2 ±3.3	1.0 ±2.8	F(1, 213.4) = 1.180, p = 0.312, n² = 0.01

Values are represented as absolute length changes [mm] within the following phases of the Stretch-shortening cycle: 100 ms before GC (PRE), eccentric (ECC) and concentric phase (CON) as mean values and SD for the muscle (LM), muscle-tendon unit (LMTU) and the tendinous tissue (LTT). Negative values indicate shortening while positive values indicate lengthening of the corresponding structure within the relevant phases. Gravity has a significant main effect on the length changes of the parallel and serial elastic structures of the muscle-tendon complex (Bolt p and F values). Effect sizes

are given by η^2. Significant differences compared to the 1g condition are marked with an asterisk (* for $p < 0.05$; ** for $p < 0.001$).
